# Targeting Astrocytic Connexin 43 Mitigates Glutamate-Driven Motor Neuron Stress in Late-Onset Spinal Muscular Atrophy

**DOI:** 10.3390/cells14231852

**Published:** 2025-11-25

**Authors:** Schahin Salmanian, Linda-Isabell Schmitt, Kai Christine Liebig, Stefanie Hezel, Andreas Roos, Ulrike Schara-Schmidt, Christoph Kleinschnitz, Markus Leo, Tim Hagenacker

**Affiliations:** 1 Department of Neurology, Center for Translational Neuro- and Behavioral Sciences (C-TNBS), University Medicine Essen, 45147 Essen, Germany; schahin.salmanian@uk-essen.de (S.S.); linda-isabell.schmitt@uk-essen.de (L.-I.S.); kaichristine.liebig@uk-essen.de (K.C.L.); stefanie.hezel@uk-essen.de (S.H.); christoph.kleinschnitz@uk-essen.de (C.K.); tim.hagenacker@uk-essen.de (T.H.); 2 Department of Pediatric Neurology, Center for Translational Neuro- and Behavioral Sciences (C-TNBS), University Medicine Essen, 45147 Essen, Germany; andreas.roos@uk-essen.de (A.R.); ulrike.schara-schmidt@uk-essen.de (U.S.-S.)

**Keywords:** calcium ion, excitotoxicity, Gap27, gap junction, motor neuron, mouse model, induced pluripotent stem cells

## Abstract

**Highlights:**

**What are the main findings?**

- Astrocytic Cx43 is markedly upregulated in late-onset SMA, in both mouse spinal cord tissue and SMN-deficient murine and human astrocytes.
- Cx43 overactivation drives excessive glutamate release and aberrant Ca^2+^ responses in motor neurons, thereby promoting excitotoxic stress.

**What is the implication of the main findings?**

- Inhibition of Cx43 hemichannels (Gap27) normalizes glutamate release from astrocytes and restores motor neuron Ca^2+^ homeostasis, demonstrating a direct functional contribution of Cx43 to SMA-related neurotoxicity.
- Targeting astrocytic dysfunction, specifically Cx43 hemichannels, represents a promising SMN-independent therapeutic strategy for late-onset SMA, complementing current SMN-restoring treatments.

**Abstract:**

5q-associated Spinal Muscular Atrophy (SMA) is a hereditary neuromuscular disorder caused by mutations in the *survival of motor neuron 1* (*SMN1*) gene, leading to progressive muscle weakness, and atrophy. While traditionally viewed as a motor neuron (MN)-specific disease, emerging evidence highlights the critical role of astrocytes, particularly in regulating extracellular glutamate and mitigating MN toxicity. Here, we investigated astrocytic gap junctions with a focus on connexin 43 (Cx43). Using in vivo and in vitro approaches—including a late-onset SMA mouse model, human-derived astrocytes, and murine astrocyte cultures—we analyzed Cx43 expression and localization via genetic modification, immunostaining, Western blotting, and quantitative PCR. Functional consequences were assessed using ex vivo spinal cord slice cultures, Ca^2+^-imaging, and glutamate release assays. We found significant Cx43 upregulation in late-onset SMA mice, as well as in SMN-deficient murine and human-derived astrocytes. Increased Cx43 expression correlated with elevated astrocytic glutamate release and MN toxicity. Ca^2+^-imaging indicated Cx43-dependent mechanisms underlying this enhanced release. Pharmacological Cx43 inhibition with Gap27 reduced glutamate release and MN Ca^2+^ responses. These findings identify astrocytic Cx43 as a contributor to glutamate-mediated MN toxicity in late-onset SMA and support growing recognition of non-neuronal mechanisms in SMA pathology.

## 1. Introduction

Spinal Muscular Atrophy (SMA) is an inherited motor neuron disorder (MND) characterized by progressive muscle atrophy caused by the loss of lower motor neurons (MNs) in the ventral horn of the spinal cord, resulting in weakness and wasting of proximal limb and respiratory muscles. The disease is triggered by homozygous loss-of-function mutations in the *survival of motor neuron 1* (*SMN1*) gene [1,2], which encodes the SMN protein, a key factor in post-transcriptional RNA processing, including mRNA splicing. Disease severity is strongly modified by the copy number of the paralogous *SMN2* gene. Patients with few *SMN2* copies typically develop severe, early onset SMA within the first months of life, while higher copy numbers lead to milder forms with later onset of symptoms [2,3,4,5,6].

The identification of the genetic basis of SMA has led to the development of therapies that restore SMN expression, with nusinersen becoming the first approved treatment in 2016 [6,7]. While SMN-enhancing drugs have significantly improved survival and motor outcomes, their therapeutic effect is limited when treatment is initiated at advanced disease stages [8,9,10,11,12]. Persistent motor deficits and the high economic burden of treatment highlight the need for complementary, SMN-independent therapeutic strategies.

Astrocytes, the predominant glial cell type in the central nervous system, have emerged as significant contributors to the pathophysiology of MND, including Amyotrophic Lateral Sclerosis (ALS) and SMA. Several studies have shown astrocytic activation preceding MN degeneration [13,14,15,16,17]. In late-onset SMA, astrocytic dysfunction has been implicated in MN death, with evidence pointing to dysregulated glutamate homeostasis as a key driver of neurotoxicity in both a late-onset SMA mouse model and patient-derived samples [16].

Astrocytes communicate through gap junctions composed of connexons, each formed by six connexin proteins [18,19]. Connexin 43 (Cx43), encoded by *GJA1*, is the predominant connexin expressed in astrocytes and plays a central role in intercellular coupling. Cx43 has been implicated in maintaining central nervous system homeostasis through multiple mechanisms, including buffering extracellular ions, synchronizing astrocytic networks, providing metabolic support to neurons, regulating the blood–brain barrier, and modulating synaptic plasticity [20,21,22]. Importantly, Cx43 has been linked to the regulation of extracellular glutamate, suggesting that astrocytic gap junctions are involved in controlling excitatory neurotransmission [23,24]. Dysregulation of astrocytic Cx43 has been associated with neurodegeneration, and its contribution to MN pathology has already been established in ALS [25].

Building on our recent findings that astrocytic activation and glutamate dysregulation drive MN death in late-onset SMA [16,17], Cx43 emerges as a promising candidate mechanism for contributing to astrocyte-mediated neurotoxicity. In the present study, we investigate the role of astrocytic gap junctions, with a focus on Cx43, in the context of late-onset SMA. To this end, we employ complementary in vitro and ex vivo approaches, including SMN-deficient human induced astrocytes (hiAstrocytes), murine astrocyte cultures, spinal cord slice preparations, and the Taiwanese mild SMA mouse model, which recapitulates the late-onset phenotype [16]. We hypothesize that astrocytic Cx43 contributes to MN degeneration in late-onset SMA and propose that targeting this protein may provide a potential SMN-independent therapeutic strategy.

## 2. Materials and Methods

### 2.1. Animals

The Taiwanese mild SMA mouse model reflecting late-onset SMA (FVB.Cg-Smn1^tm1Hung^Tg(SMN2)2Hung/J; Jackson Laboratory #005058) was obtained from the Jackson Laboratory (Bar Harbor, ME, USA) and bred in the Animal Research Facility of the University Hospital Essen (Essen, Germany). These mice are double homozygotes carrying a knockout of murine *SMN1* together with four copies of the human *SMN2* gene. Compared with wild-type FVB/N mice, they are smaller in size and exhibit reduced body weight, decreased grip strength, and hindlimb weakness. In addition, ear and tail necrosis typically occur, resulting in shortened ears and thickened, shortened tails. Previous studies have demonstrated MN loss in this model at approximately postnatal day (P) 35, defining this stage as a critical time point to investigate the role of Cx43 [16,17]. For temporal analysis, P20 was defined as the early stage, P35 as the onset of MN loss, and >P100 as the late stage of disease progression.

Wild-type (WT) FVB/N mice were used as controls and for culture experiments. For MN preparation, embryos were harvested at embryonic day (E) 14 from timed pregnant WT females. All animals were maintained under a 12 h light/12 h dark cycle with food and water provided ad libitum.

All animal experiments were performed in accordance with the institutional animal welfare guidelines of the University of Duisburg-Essen, Germany. The use of the late-onset SMA mouse model was approved by the State Office for Consumer Protection and Food Safety (LAVE), North Rhine-Westphalia, Recklinghausen, Germany (approval number 81–02.04–2020.A335).

### 2.2. Isolation of the Spinal Cord

To extract the spinal cord, WT mice were euthanized by decapitation, after deep anesthetization with isoflurane. The spine was then extracted, and the spinal cord was obtained by hydraulic extrusion. The meninges were removed, and the cord was ready for use.

### 2.3. Spinal Cord Slice Preparation

The lumbar part of the spinal cord was placed in embedding medium and snap-frozen in liquid nitrogen. The tissue was then cut into 20 µm slices using a cryotome. Every fifth section of each spinal cord was mounted on an independent microscopy slide and stored at −20 °C.

### 2.4. Culture of Spinal Astrocytes from WT Mice

The spinal cord tissue was finely diced with a razor blade and placed in a 0.25% trypsin/EDTA solution (#25200056, Thermo Fisher Scientific, Dreieich, Germany) for 30 min at 37 °C. Enzymatic digestion was halted by introducing DMEM/F12 (#210410202, Thermo Fisher Scientific, Dreieich, Germany), supplemented with 10% fetal bovine serum (FBS, #16140071, Thermo Fisher Scientific, Dreieich, Germany), to the solution. A homogenized cell solution was then produced by mechanical titration with a pipette. The solution was centrifuged down at 500 G for 5 min. The supernatant was removed, 10 mL of our culture medium (a DMEM/F12 solution containing 10% FBS and 1% penicillin/streptomycin (P/S, #15140122, Thermo Fisher Scientific, Dreieich, Germany) was added, and then transferred to a 75 cm^2^ cell culture flask (T75) and incubated at 37 °C and 5% CO_2_. The next day and for every 2 days after that, the medium was exchanged with fresh culture medium until a confluency of about 65% was reached at 10–14 days in vitro (DIV). The flask was then placed on an orbital shaker (250 rpm, 37 °C, 5% CO_2_) overnight for the microglia to detach. Subsequently, the culture medium was exchanged, and cells were detached from the cell culture flask, enumerated, and then seeded with 3500 cells per coverslip onto poly-d-lysine (PDL) (Sigma-Aldrich, Taufkirchen, Germany)-treated glass coverslips in a 24-well plate. The culture medium was again replaced in the same manner.

### 2.5. Human Tissue Samples

Human skin biopsy was performed by a physician. All participants provided informed consent, and the study was approved by the Ethics Committee of the University of Duisburg-Essen (approval number 19–9011-BO).

### 2.6. Generation of hiAstrocytes from Skin Fibroblasts

hiAstrocytes were generated from dermal fibroblasts of healthy donors as previously described [16]. In brief, fibroblasts were directly reprogrammed into induced pluripotent stem cells (iPSCs) via retroviral transduction with *Oct4*, *Sox2*, *Klf4*, and *c-Myc* (#RF101, ALSTEM, Richmond, CA, USA) in the presence of a neural induction medium. Following transfection, cells were maintained in conversion medium. Conversion medium was a solution of DMEM/F12 supplemented with 1% GlutaMAX, 1% N-2 supplement, 1% B27 supplement, 1% P/S, 20 ng/mL human fibroblast growth factor (FGF)-basic (#100-18B, PeproTech, Taufkirchen, Germany), 20 ng/mL human epidermal growth factor (EGF) (#AF-100–15, PeproTech, Taufkirchen, Germany), and 5 μg/mL heparin. Cells were passaged at a 1:2 ratio after five days to allow further multiplication and then plated after an additional 2–3 days onto PDL-coated glass coverslips.

iPSCs were subsequently cultured in iPSC medium (DMEM/F12 with 1% GlutaMAX, 1% N-2 supplement, 1% B27 supplement, 1% P/S, and 40 ng/mL human FGF-basic) until confluent. The cells were then enzymatically dissociated using Accutase and seeded into 24-well plates containing PDL-coated coverslips (4500 cells/coverslip). Differentiation into hiAstrocytes was induced using an astrocyte conversion medium (DMEM high glucose supplemented with 10% fetal bovine serum, 1% P/S, and 0.2% N-2 supplement) and maintained until approximately 80% confluency was achieved.

### 2.7. Induction of SMN Deficiency in Cultured Spinal Astrocytes

Following the replating process, small interfering (si)RNA experiments were commenced after 7 DIV. A genetic knockdown was achieved by transfecting WT murine astrocyte cultures with mouse-specific *SMN1*-siRNA (#SR408287, OriGene, Rockville, MD, USA) to induce SMN deficiency. For hiAstrocytes, human *SMN1* (#SR304480 OriGene, Rockville, MD, USA) was used. Transfection with scrambled(scr)-siRNA-FITC (#sc-36869, Santa Cruz, Dallas, TX, USA) served as our control. For this purpose, 2 h before applying siRNA to the spinal astrocytes, the medium was replaced with FBS-free medium (only DMEM/F12 containing 1% P/S).

Subsequently, 10 nM of the *SMN1*-siRNA was combined with 200 μM of SilenceMag (#SM11000, OZ Biosciences, Marseille, France) and incubated for 15 min at room temperature (RT). The solution was then added to the cells and incubated for 2 h on a magnetic plate at 37 °C and 5% CO_2_. The magnetic plate was then removed, and the cells allowed to incubate until the next day, when the siRNA medium was exchanged with fresh medium (DMEM/F12, 10% FBS, 1% P/S) and in part with 200 μM Gap27 (#E0040, Selleck Chemicals, Houston, TX, USA), a Cx43 inhibitor. The cells were maintained in culture for three more days before being used in experiments. Long exposure to Gap27 ensured Cx43 hemichannel and gap junction inhibition [26]. The efficiency of this transfection method has been previously evaluated by immunostaining for SMN-positive cells after transfection [17].

### 2.8. Immunostaining

For immunostaining, cell cultures and tissue were fixed in 4% paraformaldehyde, washed with phosphate-buffered saline (PBS), and permeabilized with 0.1 v/w Triton X-100 in PBS. The tissue was then blocked in a solution of 5% bovine serum albumin (BSA) in PBS, and the cells were treated with 1% BSA in PBS.

Primary antibodies against Cx43 (rabbit, 1:500, #SAB4501175 Sigma Aldrich, Taufkirchen, Germany), βIII-tubulin (TUJ1) (mouse, 1:500, #8578 Sigma Aldrich, Taufkirchen, Germany), non-phosphorylated neurofilament proteins (SMI-32, mouse, 1:500, #801701, BioLegend, San Diego, CA, USA), and glial fibrillary acidic protein (GFAP) (mouse, 1:500, #63893, Sigma Aldrich, Taufkirchen, Germany) were diluted in the appropriate blocking agent and incubated over night at 4 °C.

After washing, secondary antibodies (goat anti-rabbit, goat anti-mouse, 1:300, Dianova, Hamburg, Germany) and 4′,6-diamidino-2-phenylindole (DAPI, 1:500, Sigma Aldrich, Taufkirchen, Germany), diluted in blocking solution, were administered to the tissue and cells, incubated for 1.5 h at RT, then washed, covered, dried overnight, and sealed.

Images were captured utilizing a Zeiss Axio Observer.Z1 fluorescence microscope (Zeiss, Jena, Germany). The Zeiss Zen software (Version 3.4) was employed to visualize the target proteins. To ensure consistency in the analyses, all microscope settings, including laser intensity, exposure time, and contrast, were maintained at identical parameters for each protein. With the use of the ImageJ software (Version, 2.16.0, NIH, Bethesda, MD, USA), the immunoreactivity of the tagged protein in the spinal cord tissue slices was measured by marking the target area. The fluorescence intensity was then normalized against the background intensity of each area. ImageJ was used to determine the cell count of MNs and the quantity of Cx43 particles in the astrocytes.

All analyses were conducted by setting the experiment into the perspective of an appropriate control. WT mice were used as a control for SMA mice. scr.-siRNA transfected astrocytes were used as controls for SMN-deficient astrocytes.

### 2.9. Western Blots

Western blot (WB) analysis was performed to confirm the protein levels assessed through immunostaining. To achieve this, spinal cord tissue from SMA and WT mice was homogenized using RIPA buffer supplemented with a protease inhibitor cocktail (Roche, Grenzach-Wyhlen, Germany). The protein content in these lysates was quantified using a bicinchoninic acid protein assay (BCA).

A total of 10 µm of protein was applied to 4–15% TGX Stain-Free gels (Bio-Rad, Feldkirchen, Germany); subsequent protein transfer to 0.2 µm nitrocellulose membranes was achieved through a semi-dry blotting technique. Membrane images were captured for comprehensive assessment of total protein. The membranes then underwent a 10 min incubation in fast-blocking solution (Bio-Rad, Feldkirchen, Germany) with gentle agitation at RT. Following this, the membranes were incubated with primary antibodies diluted 1:8000 in blocking solution, targeting Cx43, at 4 °C overnight.

The secondary anti-rabbit antibody diluted at 1:10,000 in blocking solution and coupled to horseradish peroxidase was then introduced to the membranes after thorough washing and incubated for 90 min at RT. After another washout, an enhanced chemiluminescence substrate was added, and immunoreactivity was detected using a WB imaging system (Bio-Rad, Germany). The WB signals were analyzed with Bio-Rad imaging software (ImageLab, Version 6.1). Initially, the signal intensity of Cx43 lanes were assessed. Subsequently, the protein signal of the lanes was adjusted relative to its total protein value. Finally, the determined protein level in SMA mice was normalized against the value observed in age-matched controls. The blots and the total protein are shown in the Additional file.

### 2.10. Real-Time Quantitative Polymerase Chain Reactions

The total RNA was isolated from spinal cord samples employing Qiazol (Qiagen #79306). One microgram of each RNA sample was used for first-strand complementary DNA synthesis in a 20-μL reaction using the high-capacity cDNA RT Kit (Applied Biosystems #4368814). Expression levels of the Cx43 gene *GJA1* (forward primer: GGTGATGAACAGTCTGCCTTTCG, reverse primer: GTGAGCCAAGTACAGGAGTGTG; #MP205239, OriGene, Rockville, MD, USA) were quantified through real-time quantitative polymerase chain reaction (qPCR) analysis utilizing Power SYBR™ Green PCR Master Mix (#4,367,659, Applied Biosystems, Waltham, MA, USA). Data were normalized to a *β-actin* gene (forward primer: CATTGCTGACAGGATGCAGAAGG, reverse primer: TGCTGGAAGGTGGACAGTGAGG; #MP200232, OriGene, Rockville, MD USA).

### 2.11. Isolation and Culture of Organotypic Spinal Cord Slice Cultures from WT and Late-Onset SMA-Mice

To achieve the best resemblance of SMA pathophysiology in the mouse spinal cord and the effect of pharmacology on this tissue with the lowest burden on the animal, slice culture experiments were established. After isolating the spinal cord of late-onset SMA mice at P25(±1), the lumbar section was extracted, placed on an appropriately cut agar plate, and introduced to a vibratome filled with artificial cerebrospinal fluid (125 mM NaCl, 2.5 mM KCl, 2 mM CaCl_2_, 1 mM MgCl_2_, 25 mM NaHCO_3_, 1.25 mM NaH_2_PO_4_, 25 mM glucose). The 350 µm slices were prepared and put on inserts with a semi-permeable membrane into wells filled with a culture medium consisting of Neurobasal A (#10888022, Thermo Fisher Scientific, Dreieich, Germany) containing 1% P/S and 2% B-27 Plus Supplement (#17504044, Thermo Fisher Scientific, Dreieich, Germany). Age-matched control mice were utilized in a different experiment with the same protocol.

On 1 DIV, the culture media were replaced with fresh medium, and half of the samples were treated with 200 µM Gap27. The supernatant (SN) was collected at 7 DIV, snap-frozen in liquid nitrogen, and stored at −80 °C for experimentation purposes.

### 2.12. Isolation and Culture of Spinal Motor Neurons from Embryonic WT Mice

Embryos at E14 were harvested from the expecting mother, decapitated, fixed to a plate, and had their spinal cord removed under a microscope. The meninges were carefully removed, and the cord was kept in Neurobasal (#21103049, Thermo Fisher Scientific, Dreieich, Germany) on ice. Next, the spinal cords were introduced to our 0.25% trypsin/EDTA solution and allowed to digest at 37 °C.

After enzymatic digestion and mechanical dissociation with a pipette, the tissue was centrifuged and added to a culture medium consisting of DMEM/F12 with 1% P/S, 10% FBS, and 1:800 GlutaMAX (#35050061, Thermo Fisher Scientific, Dreieich, Germany). The cell suspension was then added to a Petri dish and incubated at 37 °C and 5% CO_2_ for 1 h. The debris was gently washed off, and the adhering cells were scraped off, added to the culture medium, and plated on PDL-coated wells (5000 cells/well). After 2 h of incubation at 37 °C and 5% CO_2_, the wells were flooded with culture medium to a total of 500 µL.

The next day, the medium was removed. A new medium was introduced, consisting of Neurobasal with 2% B-27, 1% P/S, 1:800 GlutaMAX, 1:400 ciliary neurotrophic factor (CNTF, #C-240, Alomone Labs, Jerusalem, Israel), 1:100 glial cell line-derived neurotrophic factor (GDNF, #G-240, Alomone Labs, Jerusalem, Israel), and 1 µL cytosine β-D-arabinofuranoside (AraC, #C6645, Sigma Aldrich, Taufkirchen, Germany). The medium was then changed at 8 DIV, and the culture was usable for further experimentation after 14 DIV.

### 2.13. Calcium Imaging

To determine whether inhibiting Cx43 in the mouse spinal cord had any impact on spinal MNs, the collected medium of the slice cultures was introduced to the cultured WT MN and incubated overnight at 37 °C and 5% CO_2_.

The next day, the wells were washed and introduced to 1 µM of the calcium ion (Ca^2+^) fluorescence dye Fluo-4 AM (#F14201, Thermo Fisher Scientific, Waltham, MA, USA) in FBS-free medium and incubated for 15 min. Using a Zeiss Axio Examiner fluorescent microscope, images were captured from MNs treated with SN from late-onset SMA spinal cord slices or late-onset SMA slices treated with 200 µm Gap27. The difference in basal Ca^2+^ intensity was measured using ImageJ. Therefore, the soma of MNs was selected as the region of interest (ROI).

In a separate experiment, Fluo-4 was introduced to WT MNs. The MNs were then examined under the microscope, and a video recording was started. At the one-minute mark (500 frames), the SN of SMA slices or SMA slices treated with 200 µM Gap27 was added to the cultures (point of application) and recorded for an additional three minutes. The spike amplitude of all viable cells was measured as the difference between the highest point of the first peak and baseline before application. Baseline was defined as the first 500 frames. Measurements were conducted using ImageJ Software. All experiments were performed and analyzed by investigators who were blinded to the experimental conditions.

### 2.14. Glutamate Assay

To measure the glutamate level in the supernatants of slice cultures and transfected astrocyte cultures, a glutamate assay kit (#MAK004, Sigma Aldrich, Taufkirchen, Germany) was used according to the manufacturer’s protocol. The total protein in the supernatants was measured using a BCA and normalized to the measured glutamate level and to their appropriate controls as described elsewhere to exclude density or damage-driven artifacts [16,27,28].

### 2.15. Statistical Analysis

For statistical analyses between two conditions, Student’s *t*-tests and Mann–Whitney U tests were applied; for analyses between two or more conditions, the one-way ANOVA was used. Normality was verified by applying the D’Agostino & Pearson test. Significances were defined at a value of * *p* < 0.05; ** *p* < 0.01; *** *p* < 0.001. All values are given as the mean ± standard deviation, except the area under the curve (AUC) calculation in calcium imaging data, which are presented as the 95% confidence interval (CI). All statistical analyses and their graphical presentations were made using GraphPad Prism 9. All graphical presentations of methodological approaches were conducted using biorender.com.

## 3. Results

### 3.1. Spinal Cord Tissue Exhibited Increased Expression of Cx43 in Late-Onset SMA Mice

**Figure 1:**
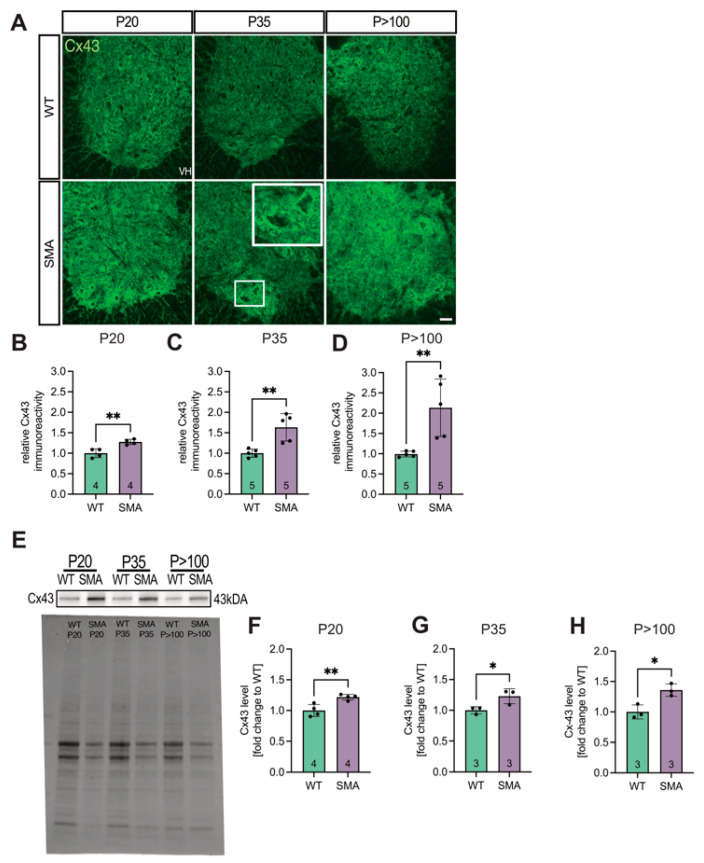
Late-onset SMA spinal cord analyses showed an increased expression of Cx43 compared to WT. (**A**) Spinal cord slices of SMA and WT mice at ages P20, P35, and *p* > 100 (*n* = 4–5 animals) were stained for Cx43 (green). (**B**–**D**) Imaging studies showed an increased expression of Cx43 in SMA at P20 (** *p* = 0.0035). This increased expression was also visible at P35 and *p* > 100 (** *p* = 0.0094 and *p* = 0.0071, respectively). Higher accumulation of Cx43 was evident around motor neurons, particularly in SMA, pointed out in the SMA P35 image. Scale bar 20 μm. (**E**) For WB studies, lumbar spinal cord tissue of *n* = 3–4 animals was used. Results were then adjusted to the total protein value. (**F**–**H**) At P35 (* *p* = 0.0309) and *p* > 100 (** *p* = 0.0037), Cx43 expression was increased in SMA compared to WT. At P20, we found no difference in the Cx43 expression (*p* = 0.1115). Analysis was conducted using unpaired Student’s *t*-tests. Full blots are shown in the Additional file. Abbreviations: SMA, spinal muscular atrophy; WB, Western blot; WT, wild-type; P, postnatal day.

To examine Cx43 expression in situ, lumbar spinal cord sections from late-onset SMA and WT mice were immunostained and analyzed (Figure 1A). Quantification of total Cx43 immunoreactivity within the ventral horn, normalized to WT levels, revealed significantly increased expression in late-onset SMA tissue at P20 (1.25-fold, *p* = 0.0035), P35 (1.59-fold, *p* = 0.0094), and *p* > 100 (2.3-fold, *p* = 0.0071) (Figure 1B–D).

These findings were confirmed by Western blot analysis (Figure 1E–H, and Supplementary File Figure S1A–D).

### 3.2. Cx43 mRNA Expression Is Post-Transcriptionally Modulated

**Figure 2:**
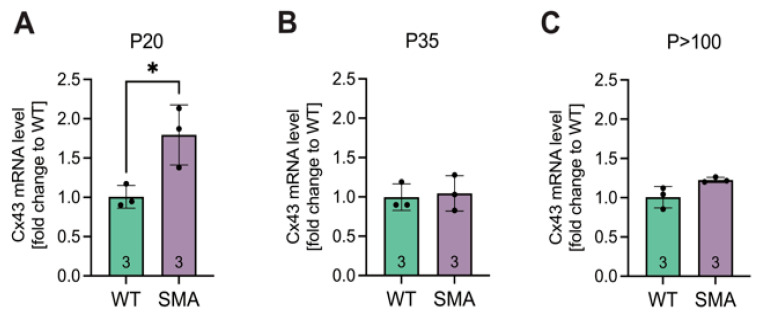
qPCR analyses showed an increase in Cx43 mRNA expression early in the pathogenesis. (**A**) Whole lumbar spinal cord tissue of SMA and WT mice at P20, P35, and *p* > 100 were prepared, and qPCR analyses were performed. The results suggested an increase in mRNA expression at P20 in SMA compared to WT (* *p* = 0.029). (**B**,**C**) No significant difference was visible between SMA and WT at later stages (P35, *p* = 0.9485; *p* > 100, *p* = 0.0542). *n* = 3 individual animals, analysis was conducted using unpaired Student’s *t*-tests. Abbreviations: SMA, spinal muscular atrophy; qPCR, quantitative polymerase chain reaction; WT, wild-type; P, postnatal day.

To investigate whether Cx43 expression is transcriptionally regulated in late-onset SMA pathology, qPCR analysis was performed on lumbar spinal cord tissue. Cx43 mRNA levels were significantly elevated 1.8-fold in the spinal cord tissue of late-onset SMA mice compared to WT at P20 (*p* = 0.029) (Figure 2A). In contrast, no significant differences were observed at P35 (*p* = 0.9485) or P > 100 (*p* = 0.0542) (Figure 2B,C).

### 3.3. SMN Deficiency Increased Astrocytic Cx43 Expression

**Figure 3:**
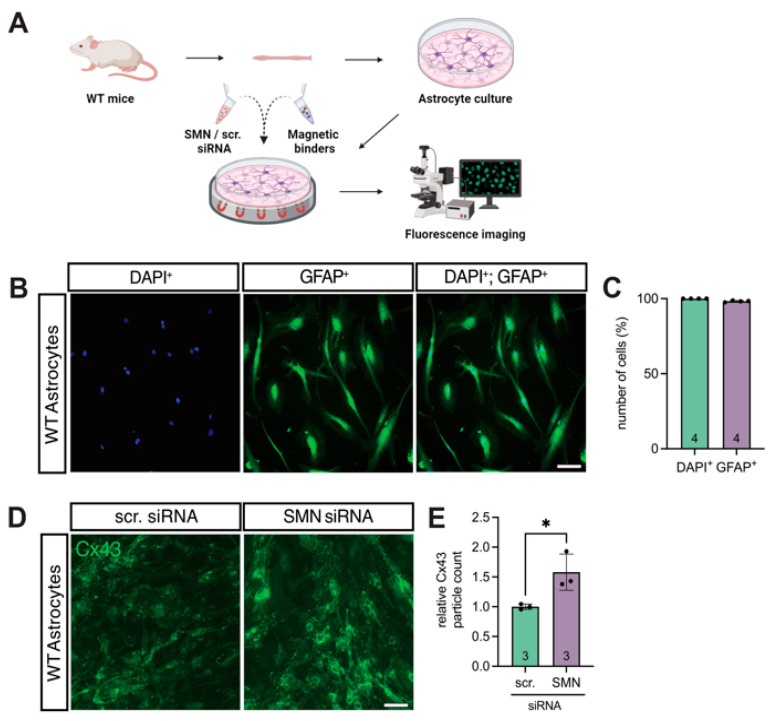
Overexpression of Cx43 in a model of in vitro-induced SMN-deficiency in astrocytes. (**A**) Astrocytic cultures were prepared from the lumbar part of the spinal cord of WT mice and transfected with SMN-siRNA to induce SMN-deficiency. Appropriate control cultures were transfected with scr-siRNA and fluorescence imaging was conducted (created with bioRender.com). (**B**,**C**) GFAP (green) and DAPI (blue) staining of the same cultures demonstrated an astrocyte proportion of >98% compared to all viable cells; *n* = four individual cultures for each of the three animals, scale bar 50 μm. (**D**,**E**) Immunostaining of Cx43 (green) in transfected astrocytes. Analysis showed a 1.6-fold increase in Cx43 expression in SMN-deficient cells compared to the control (* *p* = 0.03; *n* = three independent experiments for each of the three individual animals, analysis was conducted using unpaired Student’s *t*-tests, scale bar 50 μm. Abbreviations: DAPI, 4′,6-diamidino-2-phenylindole; GFAP, glial fibrillary acidic protein; siRNA, small interfering RNA; SMN, survival of motor neuron; WT, wild-type.

To assess whether SMN deficiency affected Cx43 expression in spinal astrocytes, primary WT astrocyte cultures were transfected with SMN-targeting siRNA, while cultures transfected with scr-siRNA served as controls (Figure 3A).

The astrocytic purity of the cultures was verified by GFAP immunostaining, revealing that >98% of all DAPI^+^ cells were GFAP^+^ (Figure 3B,C). Immunolabeling for Cx43 followed by quantitative particle analysis demonstrated a 1.6-fold increase in Cx43 expression in SMN-deficient astrocytes compared to control cells (*p* = 0.0298) (Figure 3D,E).

### 3.4. Translational Validation of Cx43 Dysregulation Using a Human iPSC-Based Model

To further explore the contribution of Cx43 dysregulation in late-onset SMA, we employed an iPSC-based human model. hiAstrocytes were generated and transfected with SMN1-specific siRNA to mimic SMN deficiency observed in late-onset SMA mice.

**Figure 4:**
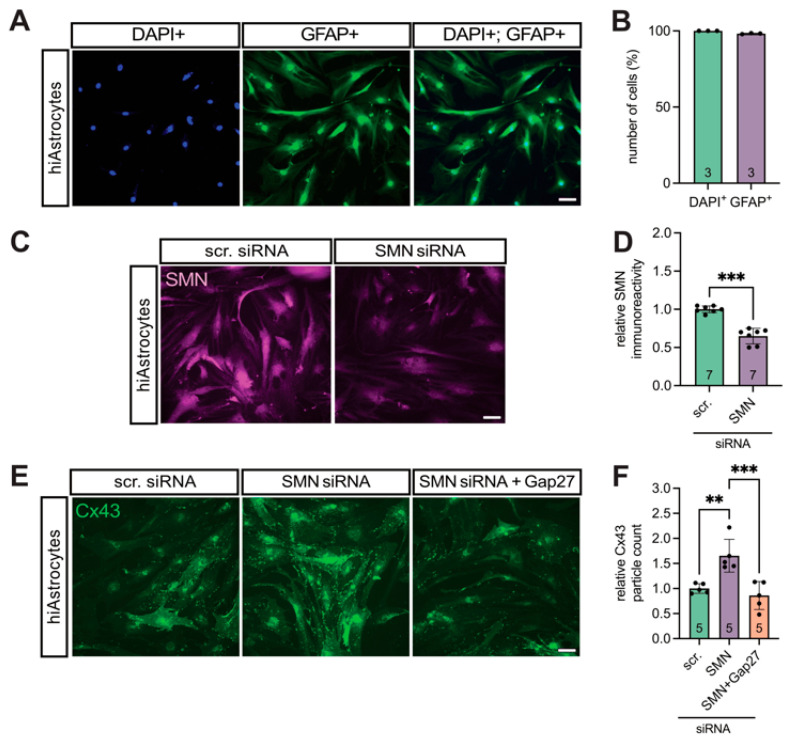
SMN deficient hiAstrocytes showed a higher Cx43 expression compared to the control, reversible by Gap27. (**A**,**B**) Culture clarity and iPSC induction success were proven by GFAP staining, showing >98% astrocytes in the cultures. (**C**,**D**) hiAstrocytes were transfected with SMN-siRNA to induce SMN deficiency. Appropriate controls were transfected with scr-siRNA. Staining for SMN showed an effective decrease compared to the control (*** *p* = 0.0006). (**E**,**F**) Staining for Cx43 showed an increased expression after SMN-knockdown (** *p* = 0.02), which was reduced after treatment with the Cx43 inhibitor Gap27 (*** *p* = 0.0009). *n* = three to seven independent experiments, analysis was conducted using unpaired Student’s *t*-tests and ANOVA, scale bar 50 μm. Abbreviations: DAPI, 4′,6-diamidino-2-phenylindole; E, embryonal day; SMA, spinal muscular atrophy; WT, wild-type.

Astrocytic identity and culture purity were confirmed by GFAP immunostaining, showing that >98% of all DAPI^+^ cells were GFAP^+^ (Figure 4A,B).

Transfection efficiency was verified by SMN immunostaining, revealing a significant reduction in SMN expression in SMN-siRNA-treated cells compared to scr-siRNA controls (0.82-fold, *p* = 0.006) (Figure 4C,D).

SMN-deficient hiAstrocytes exhibited a 1.7-fold increase in Cx43 expression relative to the controls (*p* = 0.02), which was reduced upon treatment with the Cx43 inhibitor Gap27 (0.5-fold, *p* = 0.007) (Figure 4E,F).

### 3.5. Cx43 Inhibition Reduces SMA-Associated Calcium Levels in MNs

**Figure 5:**
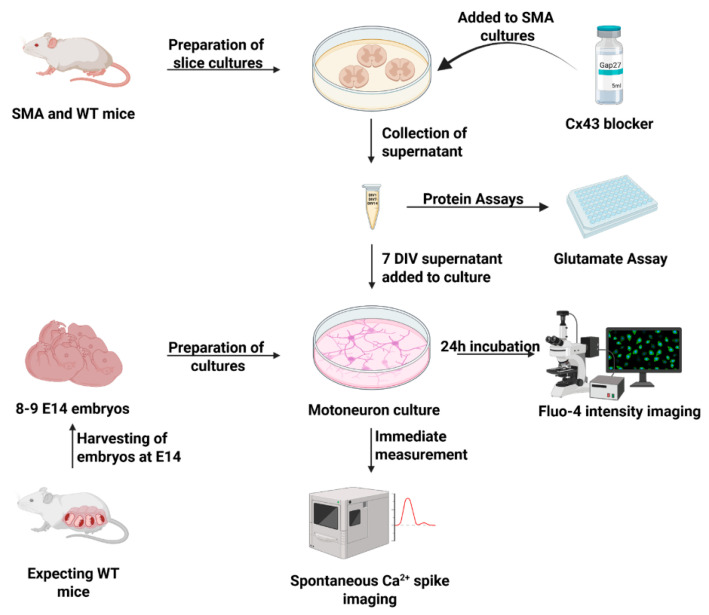
Preparation of organotypic spinal cord slice cultures as an in vitro model of SMA. Slice cultures from WT and SMA mice were prepared. Supernatant was removed at 1 DIV, and half of the SMA cultures were treated with 200 µM Gap27. Supernatant was again removed at 7 DIV and at 14 DIV. Embryonal motor neuronal cultures were prepared, and after maturation, the 7 DIV supernatant was introduced to the cultures. After 24 h incubation, the Ca^2+^ intensity was measured. In another experiment, the Ca^2+^ spike amplitude was measured immediately after application of 7 DIV supernatants. All supernatants were assayed for glutamate with glutamate assays (created with bioRender.com). Abbreviations: DIV, days in vitro; SMA, spinal muscular atrophy; E, embryonal day; WT, wild-type.

To investigate the functional impact of pathological Cx43 expression on spinal MNs, organotypic spinal cord slice cultures and primary MN cultures were prepared. MNs were subsequently incubated with supernatants (SNs) collected from treated and untreated spinal cord slice cultures of late-onset SMA mice and untreated WT mice (Figure 5).

**Figure 6:**
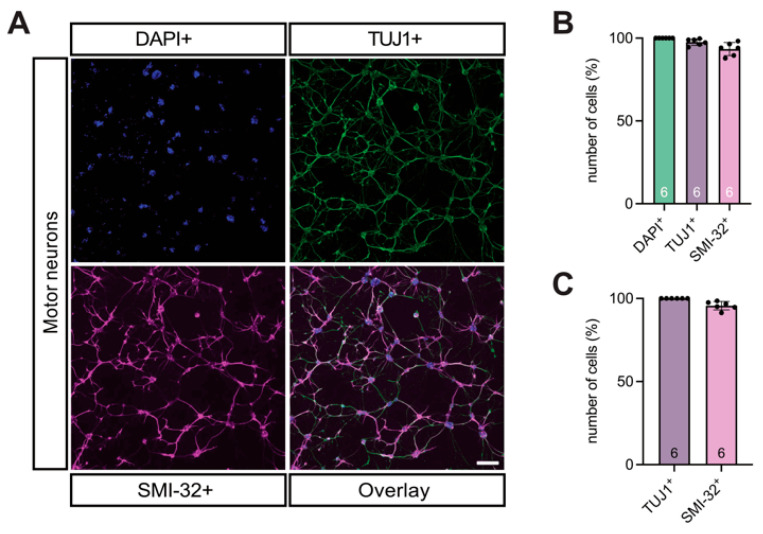
Clarity of motor neuron cultures were tested using TUJ1 and SMI-32 staining. (**A**–**C**) DAPI (blue), TUJ1 (green) for neurons, and SMI-32 (magenta) for MN staining of the neuronal cultures demonstrated that all viable cells in the culture were comprised of neurons (>97%) and motor neurons (>93%). MNs made up >95% of all neurons in the culture. *n* = six independent experiments from six individual cultures, each prepared from 8–9 WT E14 embryos, scale bar 100 μm. Abbreviations: DAPI, 4′,6-diamidino-2-phenylindole; E, embryonal day.

Immunocytochemical characterization revealed that >97% of the cultured cells were TUJ1^+^ neurons, of which >93% were SMI-32^+^ MNs, confirming a motor neuron purity of >95% (Figure 6A–C).

After overnight incubation of MNs with SN collected from 7 DIV spinal cord slice cultures of WT, late-onset SMA, and SMA treated with the Cx43 inhibitor Gap27 (SMA + Gap27), intracellular Ca^2+^ levels were assessed using Fluo-4 staining.

**Figure 7:**
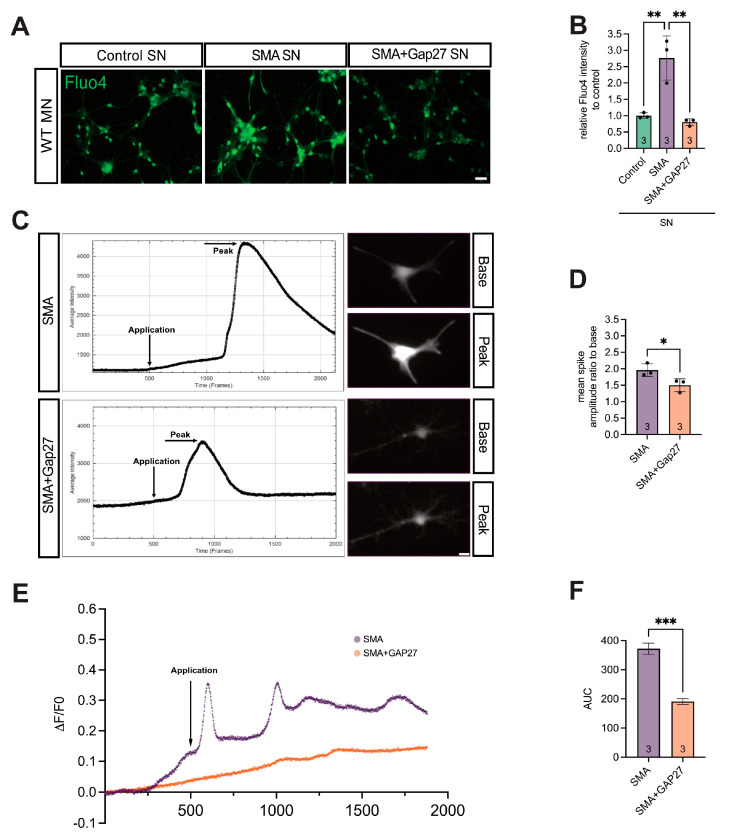
Inhibition of Cx43 in SMA slice cultures led to significantly lower WT-like Ca^2+^ response in murine MN. (**A**,**B**) 24 h incubation of WT MNs with the SN from SMA slice cultures (SMA) caused a significant increase in the Ca^2+^ response compared to incubation with the SN from WT mice slice culture (Control) (** *p* = 0.0076), measured by Fluo-4 staining to visualize Ca^2+^ processes (green). MNs incubated with SMA slice culture SN that had Cx43 inhibited (SMA + Gap27) caused a significant decrease in the Ca^2+^ response compared to incubation with the untreated SMA supernatant (** *p* = 0.002). SMA + Gap27 SN showed a result comparable to the control (*p* = 0.82). *n* = three independent experiments, scale bar 100 μm. (**C**,**D**) After acquiring the results from (**A**), the change in the spontaneous Ca^2+^ response in MNs to SN of SMA or SMA + Gap27 slice cultures was measured. Acute introduction of SMA + Gap27 slice culture supernatant to WT MNs (application at 500 frames = 1 min) showed a significant decrease in Ca^2+^ spike amplitude compared to treatment with the SMA supernatant, visualized with Fluo-4 staining in all vial cells (* *p* < 0.05). (**E**) ΔF/F0 calcium imaging traces of SMA and SMA + Gap27 MN. (**F**) AUC of ΔF/F0 calcium imaging traces displayed in mean with 95% CI. MN treated with supernatant (SMA + Gap27) showed a reduced AUC (*** *p* < 0.001). *n* = three cell cultures per condition, each consisting of six embryonic mice, with at least 30 cells/experiment/condition measured. Scale bar 10 μm, analysis was conducted using unpaired Student’s *t*-tests and ANOVA. Abbreviations: MN, motor neuron; SMA, spinal muscular atrophy; SN, supernatant; WT, wild-type.

Microscopic analysis revealed a 2.8-fold increase in Ca^2+^ response in MNs incubated with SMA SN compared to WT SN (*p* = 0.0076). Treatment of SMA slice cultures with Gap27 significantly reduced the Ca^2+^ response in MNs by 0.29-fold compared to SMA SN (*p* = 0.002), restoring levels comparable to those observed in MNs exposed to WT SN (*p* = 0.82) (Figure 7A,B).

In addition, short-term exposure of Fluo-4-loaded MNs to SN from SMA or SMA + Gap27 slice cultures, followed by Ca^2+^ imaging, revealed a significant 0.76-fold reduction in spontaneous spike amplitude in MNs treated with SMA + Gap27 SN compared to SMA SN (*p* = 0.0156) (Figure 7C,D). The ΔF/F0 was calculated, and traces for SMA and SMA + Gap27 were graphically shown (Figure 7E). AUC was reduced in SMA + Gap27 traces compared to SMA traces (*p* < 0.001) (Figure 7E).

### 3.6. SMA Cultures Showed Elevated Glutamate Levels, Reversed by Inhibiting Cx43

**Figure 8:**
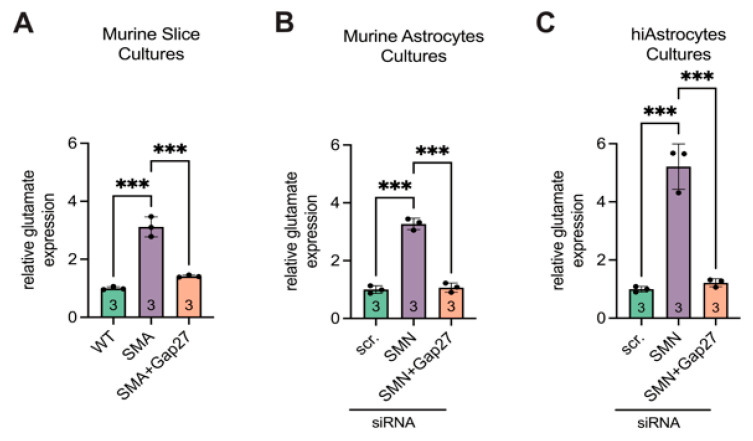
Glutamate assay showed increased expression in SMA, reversible by inhibiting Cx43. (**A**) Slice cultures showed higher glutamate levels in SMA compared to WT (*** *p* < 0.001). Gap27 treatment (200 µM) in SMA resulted in a decrease in glutamate levels (*** *p* < 0.001) to the level of WT (*p* = 0.1). (**B**) Murine cell cultures also showed higher glutamate levels when SMN was knocked down (*** *p* < 0.001), with the levels decreasing with 200 µM Gap27 (*** *p* < 0.001) to the level of WT (*p* = 0.85). (**C**) Similarly, hiAstrocytes showed increased glutamate levels when SMN was knocked down (*** *p* < 0.001). This significantly decreased after treatment with 200 µM Gap27 (*p* < 0.001) to the level of the control (*p* = 0.92). Analysis was conducted using unpaired Student’s *t*-tests. Abbreviations: SMA, spinal muscular atrophy; SMN, survival of motor neuron; WT, wild-type. hiAstrocytes, human induced astrocytes.

Glutamate assay analysis revealed a 3.1-fold increase in extracellular glutamate levels in late-onset SMA spinal cord slice cultures compared to WT (*p* < 0.001). Treatment of SMA slice cultures with the Cx43 inhibitor Gap27 significantly reduced glutamate levels (*p* < 0.001), restoring them to values comparable to WT (*p* = 0.1) (Figure 8A).

Supernatants from the siRNA-transfected murine astrocyte cultures showed consistent results. SMN-deficient astrocytes exhibited a 3.3-fold increase in glutamate levels compared to the scr-siRNA controls (*p* < 0.001), whereas Gap27 treatment normalized glutamate concentrations to control levels (*p* < 0.001 vs. SMA; *p* = 0.85 vs. scr-siRNA) (Figure 8B).

Similarly, in hiAstrocyte cultures, siRNA-mediated SMN knockdown led to a 5.5-fold increase in glutamate levels (*p* < 0.001). Gap27 treatment significantly reduced glutamate release (*p* < 0.001), restoring levels to those observed in scr-siRNA-treated hiAstrocytes (*p* = 0.92) (Figure 8C).

## 4. Discussion

This study identified astrocytic Cx43 as a contributor of glutamate-driven MN loss in late-onset SMA. We demonstrated that SMN deficiency leads to a pronounced upregulation of Cx43 in spinal astrocytes, both in a murine and in human iPSC-derived model. Elevated Cx43 expression was accompanied by increased extracellular glutamate and abnormal MN Ca^2+^ responses, indicating enhanced excitotoxic stress. Pharmacological inhibition of Cx43 with the hemichannel blocker Gap27 effectively normalized glutamate levels and restored MN Ca^2+^ signaling to control values, suggesting a direct functional link between astrocytic Cx43 and MN excitotoxicity in SMA.

Our findings highlight the importance of non-neuronal mechanisms in SMA pathogenesis. Although SMA is considered an MND caused by loss of SMN protein, growing evidence indicates that astrocytes critically modulate disease progression. Previous work has shown astrocytic activation and glutamate dysregulation preceding MN degeneration in late-onset SMA mice [16,17]. The present data extend these findings by identifying Cx43 as a central molecular contributor to this glial dysfunction.

Cx43 is the predominant connexin in astrocytes and forms both gap junctions and hemichannels that regulate intercellular signaling, ion homeostasis, and neurotransmitter clearance [22,24]. Under pathological conditions, excessive opening of Cx43 hemichannels results in the uncontrolled release of glutamate, ATP, and other neuroactive molecules, thereby amplifying excitotoxicity and inflammation [29]. Our results indicate that late-onset SMA condition promotes such aberrant Cx43 activity, leading to impaired glutamate buffering and excitotoxic stress on MNs. The reversal of these effects by Gap27 strongly indicates that Cx43 hemichannels are functionally involved in the astrocyte-mediated toxicity observed in late-onset SMA.

Gap27 is a specific mimetic peptide of Cx43 [30]. Its selectivity on hemichannels and gap junctions is time-dependent, rapidly inhibiting Cx43 hemichannels while gap junction inhibition needs longer exposure [26]. Here, Gap27 was applied to spinal cord slice cultures and spinal astrocytes until 7 DIV and 1 DIV, respectively, suggesting gap junction inhibition.

Cx43 protein has a rapid turnover rate with a half-life of 1–2 h [31], which is influenced by post-transcriptional modifications [32]. These modifications can be altered due to various stimuli linked to different diseases [33]. Prolonged exposure to Cx43 inhibitors like Gap27 may therefore be necessary to induce extended changes in Cx43 hemichannel regulation. Bioavailability and metabolism rates of both Cx43 and inhibitors like Gap27 therefore need to be considered in in vivo studies and clinical trials. Furthermore, at the molecular level, the early but transient increase in Cx43 mRNA and sustained protein overexpression suggest post-transcriptional dysregulation. This pattern aligns with the established function of SMN in RNA processing and transport [34,35] and supports the hypothesis that SMN deficiency interferes with the post-transcriptional regulation of astrocytic genes. Such mechanisms may contribute to the persistent elevation of Cx43 protein seen at symptomatic and late disease stages.

The upregulation of astrocytic Cx43 is not unique to SMA and has been described in other neurodegenerative and neuromuscular disorders, including ALS, Duchenne Muscular Dystrophy, and Multiple Sclerosis [15,25,36]. In ALS, Cx43-dependent hemichannel opening has been shown to promote MN degeneration, and its inhibition mitigates excitotoxic damage [25]. Our study provides converging evidence that similar mechanisms operate in late-onset SMA, reinforcing the concept that Cx43-mediated astrocytic dysfunction is a common pathway contributing to MN loss across distinct MNDs.

Importantly, our experiments using human SMN-deficient astrocytes reproduced the findings obtained in mouse models, underscoring the translational relevance of this mechanism. Gap27 treatment normalized glutamate release in both systems, suggesting that pharmacological modulation of Cx43 may represent a potential therapeutic strategy. Such approaches could be especially valuable for late-onset SMA patients who are diagnosed at advanced stages, when SMN-enhancing therapies alone show limited efficacy [37]. Recently published studies suggest that in both SMA patients and human cells with SMN deficiency, reduced EAAT1 expression is not corrected solely by increasing SMN levels but remains unchanged [16,38]. This indicates that EAAT1 may constitute an SMN-independent therapeutic target and that astrocyte-mediated glutamate toxicity may persist despite SMN restoration. Moreover, since astrocytes under SMA conditions have been shown not only to exhibit diminished glutamate uptake but also to actively secrete glutamate [16,17], adjunct treatment with Cx43 inhibitors could further mitigate disease pathogenesis.

Nevertheless, several limitations must be acknowledged. The study relied primarily on ex vivo and in vitro systems, which, while allowing for controlled mechanistic analysis, may not have fully captured the complexity of the in vivo spinal environment. In addition, although Gap27 efficiently inhibited pathological Cx43 activity in culture, its limited bioavailability and tissue penetration pose challenges for clinical application [39]. Future work should therefore employ conditional, astrocyte-specific Cx43 knockout models and evaluate clinically tested gap junction modulators such as Tonabersat [40,41,42] in different phenotype-related SMA models to determine their therapeutic potential.

Beyond glutamate regulation, Cx43 hemichannels also control the release of ATP and cytokines [43,44], which may further exacerbate neuroinflammation and MN injury. Thus, astrocytic Cx43 likely contributes to late-onset SMA pathology through multiple converging mechanisms that warrant deeper investigation.

## 5. Conclusions

This study identified astrocytic Cx43 as regulator of glutamate-mediated MN stress in late-onset SMA and demonstrated that its inhibition mitigates excitotoxicity. These findings expand current concepts of late-onset SMA pathogenesis beyond SMN deficiency and underscore the therapeutic potential of targeting astrocytic dysfunction as a complementary strategy to existing SMN-enhancing treatments.

## Data Availability

The datasets used and/or analyzed during the current study are available from the corresponding author on reasonable request.

## Supplementary Materials

The following supporting information can be downloaded at: https://www.mdpi.com/article/10.3390/cells14231852/s1, Figure S1: Images A to D contain full Western Blots stained for Cx43 and total proteins of the lumbar spinal cord, with each showing the results from one mouse per condition (SMA P20, SMA P35, SMA P > 100, WT P20, WT P35 and WT P > 100). The total protein was used for normalization. The Cx43 bands used for analysis are highlighted with red boxes. Abbreviations: SMA, spinal muscular atrophy; P, postnatal day; WT, wildtype; Cx43, connexin 43.

## Abbreviations

The following abbreviations are used in this manuscript:

ALS	amyotrophic lateral sclerosis
ATP	adenosine triphosphate
BCA	bicinchoninic acid protein assay
Ca^2+^	calcium ion
Cx43	connexin 43
DAPI	4′,6-diamidino-2-phenylindole
DIV	days in vitro
DMD	Duchenne muscular dystrophy
E	embryonal day
EGF	epidermal growth factor
ELISA	enzyme-linked immunosorbent assay
FBS	fetal bovine serum
FGF	fibroblast growth factor
iPSC	induced pluripotent stem cells
GFAP	glial fibrillary acidic protein
GJA1	gap junction alpha-1
hiAstrocytes	human induced astrocytes
late-onset SMA	late-onset spinal muscular atrophy
P	postnatal day
PBS	phosphate-buffered saline
PDL	poly-d-lysine
qPCR	quantitative polymerase chain reactions
RT	room temperature
scr	scrambled
SMA	spinal muscular atrophy
SMI-32	non-phosphorylated neurofilament H
SMN1	survival of motor neuron-1
SMN2	survival of motor neuron-2
SMN	survival of motor neuron
TUJ1	βIII-tubulin
WB	Western blot
WT	wild-type
