# Supplementary material for: Targeting Astrocytic Connexin 43 Mitigates Glutamate-Driven Motor Neuron Stress in Late-Onset Spinal Muscular Atrophy
Source: Cells. 2025 Nov 25;14(23):1852. doi: 10.3390/cells14231852 (PMC12691144; doi:10.3390/cells14231852)
Supplement: Supplementary file 1 [file cells-14-01852-s001.zip › cells-3989512-supplementary.pdf]

Targeting Astrocytic Connexin 43 Mitigates Glutamate-Driven Motor Neuron Stress in Late-Onset Spinal Muscular Atrophy

Schahin Salmanian[1], Linda-Isabell Schmitt[1], Kai Christine Liebig[1], Stefanie Hezel[1], Andreas Roos[2], Ulrike Schara-Schmidt[2], Christoph Kleinschnitz[1], Markus Leo[1,*] and Tim Hagenacker[1]

[1]Department of Neurology and Center for Translational Neuro- and Behavioral Sciences (C-TNBS), University Medicine Essen, Essen, Germany

[2]Department of Pediatric Neurology, Center for Translational Neuro- and Behavioral Sciences (C-TNBS), University Medicine Essen, Essen, Germany

* Correspondence: markus.leo@uk-essen.de; Tel.: +49 201 / 723 82366

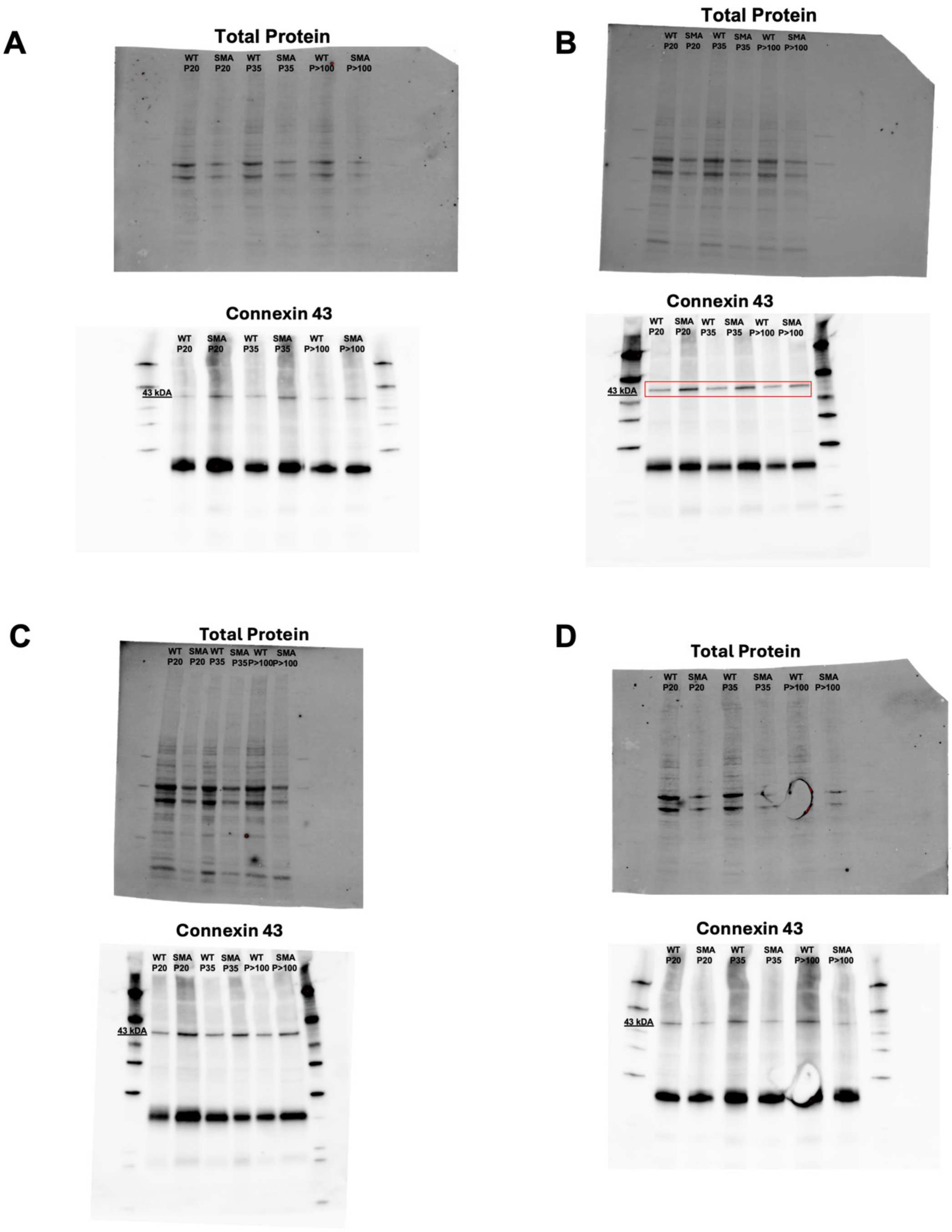

Figure S1: Images A to D contain full Western Blots stained for Cx43 and total proteins of the lumbar spinal cord, with each showing the results from one mouse per condition (SMA P20, SMA P35, SMA P>100, WT P20, WT P35 and WT P>100). The total protein was used for normalization. The Cx43 bands used for analysis are highlighted with red boxes. Abbreviations: SMA, spinal muscular atrophy; P, postnatal day; WT, wildtype; Cx43, connexin 43
